# Papillary breast lesions diagnosed by percutaneous needle biopsy: management approach

**DOI:** 10.3332/ecancer.2019.902

**Published:** 2019-02-05

**Authors:** Jorge Andrés Pérez Fuentes, Carmen Elena Marín Martínez, Ana Karina Ramírez Casadiego, Víctor Francisco Acosta Freites, Víctor Arturo Acosta Marín, Ariana Cecilia Ruiz Castellano

**Affiliations:** Centro Clínico de Estereotáxia—Ceclines, Caracas 1050, Venezuela

**Keywords:** percutaneous breast biopsy, percutaneous core-needle biopsy, papilloma, breast, core needle biopsy, vacuum-assisted breast biopsy, papillary carcinoma, breast biopsy, upgrade, breast papilloma management

## Abstract

Papillary breast lesions are a heterogeneous group of neoplasms of diverse imagenological, clinical and morphological presentation that display different behaviour, prognosis and, therefore, controversial diagnosis and management. The aim of this study is to propose an algorithm for the management of mammary lesions.

**Materials and methods:**

We show a retrospective review of breast imaging reporting, percutaneous needle biopsy information, histological-pathological reports and subsequent management.

**Results:**

A total of 7,920 biopsies were reviewed. Only 136 biopsies from 130 patients with papillary lesions met the inclusion criteria. There was a correlation between the pathologic findings from percutaneous biopsy and the final surgical histologic result in patients with surgery recommendation in all but 2 (2.12%) cases in which the surgery results were upgraded to a malignant disease.

**Conclusions:**

The algorithm proposed in this paper for the management of mammary lesions significantly reduces the possibilities of upgrading and favours decision making between follow-ups or surgery in patients with papillary lesions of the breast.

## Introduction

Papillary breast lesions (PBLs) represent a heterogeneous group of neoplasms [1–7] with diverse morphological appearance, clinical presentation, imaging findings and clinical behaviour [5]. Malignant lesions may have different prognosis [4]. PBLs can be subcategorized an intraductal papilloma (IDP), papilloma with atypical hyperplasia or atypical papilloma, papilloma with ductal carcinoma *in situ* (papilloma with DCIS), papillary DCIS (PDCIS), encapsulated papillary carcinoma and papillary carcinoma [5–8]. PBLs may present as solitary or multiple lesions, with or without nipple discharge, may have microcalcifications and may present either as a palpable mass or as a clinically occult lesion, incidentally discovered by imaging method [5].

Although most of the PBLs are benign, a percentage of them may show atypical histological features or may be malignant. Although not all papillary lesions are identified by imaging methods, they are usually detected by these methods. Sonographically, not all PBLs show as a solid intracystic or intraductal lesion. Some of them are seen as solid nodules not distinguishable from other solid lesions of the breast. If PBL is identified by imaging methods neither mammography nor ultrasound can easily distinguish between malignant or benign lesions [1].

Initial diagnosis of PBLs by image-guided percutaneous needle biopsy has furthermore replaced surgical excision in many institutions all around the world [1, 9]. However, the study and adequate categorization of PBLs with percutaneous needle biopsy still remain controversial [9]. This is partly due to the probability of tissue fragmentation. The fragmentation inherent in this type of lesion sometimes results in a disaggregated material of difficult interpretations not only of the assessment of the lesion´s intrinsic morphological findings but also in its real relation with the adjacent breast parenchyma, which represents the greatest difficulty for the adequate categorization of a benign versus malignant, noninvasive or invasive papillary lesions. Despite these disadvantages, recently imaging-guided breast biopsy has been increasingly used in the diagnosis of PBLs benign, atypical or malignant [2, 5, 10].

The standard management for malignant and atypical papillary lesions is surgical excision [11]. However, subsequent management of a benign PBL is controversial. Some studies consider intraductal papillomas to be benign lesions without malignant potential or risk for developing a subsequent carcinoma. Others recommend the surgical removal of all papillary lesions, including benign due to a high association between breast cancer and papillomas [2, 11]. This recommendation is based on the probability of up to 24.5% of the benign PBLs upgrade to malignant [5].

Recently, some authors recommended that benign papillary lesions diagnosed by core needle biopsy (CNB) might not require immediate excision, but may be safely managed with imaging follow-up [10] for at least 5 years [12] rather than with surgical excision.

The accuracy of imaging-guided percutaneous breast biopsy depends on the system used, type and gauge of the needle, quantity and adequate processing of material obtained, and finally, the correlation between the radiological image and the pathological result [13, 14].

The aim of this study is to evaluate the algorithm management for PBLs diagnosed by percutaneous needle biopsy. This algorithm is based on mammographic and/or ultrasonographic images, lesion size, percentage of lesion removed by biopsy, cannula or needle type and gauge, pathological review, concordance of the image—pathological evaluation and lesion upgrade.

## Materials and methods

### Patients and samples

We display a retrospective review from the Centro Clínico de Esterotaxia database in Caracas, Venezuela. This study was approved by the institutional review board. Patient’s informed consent was not required. The inclusion criteria were consecutive patients who underwent image-guided percutaneous needle biopsy of suspicious breast lesions that resulted PBL´s between March 1996 and December 2017. The exclusion criteria were patients with invasive papillary carcinoma, for whom chemotherapy/hormone replacement therapy was ongoing or were not treated in our institution (surgery or follow-up). The data collection process was done with Rotator Survey, a software application for the Windows environment (http://rotatorsurvey.com).

Medical records were reviewed for demographics data including clinical and radiographic characteristics, breast imaging reporting and data system (BI-RADS) categories [15], percutaneous needle biopsy information (image-guided method, radiographic size of the lesion and needle gauge), histological-pathological reports and subsequent management (follow-up or surgery).

Percutaneous needle biopsies were performed with either mammographic (stereotactic) or ultrasound guidance based on the method that allows the best visualisation of the lesion. Other considerations included imaging characteristics, lesion location within the breast and radiologist’s preference.

The stereotactic biopsies were performed using a prone stereotactic biopsy table unit Mammotest (Mammovision Fisher, Denver, CO or Mammovision Siemens, Germany). All of them were performed with the vacuum-assisted device either with a Mammotome, 11G or 8G probe (Mammotome, Biopsies/Ethicon Endo-Surgery, Cincinnati, OH) or with a Suros probe 12G or 9G (ATEC, Hologic, Inc. Marlborough, MA).

Ultrasonographic guidance was performed using different brand units with 7.5-MHz transducers or more, linear, multi-frequency or matrix array. These biopsies were performed using any of the following devices: Mammotome Breast Biopsy System 8G/11G; EnCor Breast Biopsy System 7G/10G (SenoRx Inc., Irvine, CA); Suros Surgical’s ATEC Saphire system 9G/12G or 14G needle with automatic or semi-automatic biopsy gun from different brands.

**Figure 1.**
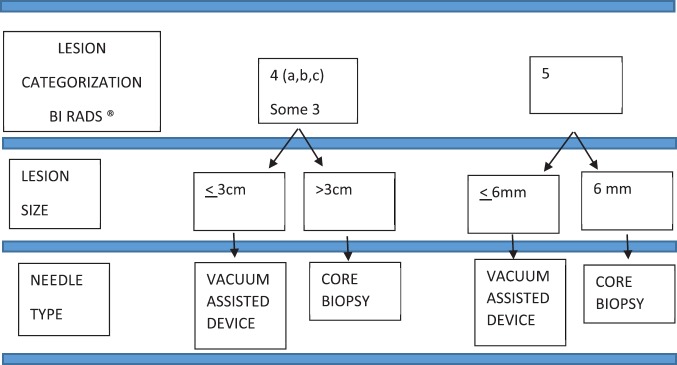
Ultrasound biopsy algorithm.

Ultrasound biopsies were performed following the algorithm described below (Figure 1). For lesions BI-RADS category 4A, B or C [15], a vacuum-assisted breast biopsy was used if it measured 3 cm or less (palpable or nonpalpable mass, complex lesions, cysts with mural thickening, intramural nodules or thick septations, intracystic mass or with atypical architecture), and CNB method if it measured more than 3 cm in size. A similar procedure was applied for lesions considered probably benign (BI-RADS 3) with recommendation for diagnostic confirmation by histological analysis instead of a follow-up.

Before the fourth Edition of BI-RADS published in 2003 [16], we classified BI-RADS 4 lesions in B4 low and B4 high. For the purpose of our study, we re-defined the lesions B4 low like BI-RADS category 4A, B and the lesions B4 high like BI-RADS category 4C.

For lesions classified BI-RADS, five vacuum-assisted breast biopsy was used if its size was 6 mm or less depending on the shape and features, and CNB was used if its size was 6 mm or more.

In all cases, the goal was the complete removal of the lesion found at imaging. In those cases in which complete removal of the lesion was achieved, a localising clip was placed to ensure identification of the tumour bed or in cases where the patient was going to receive primary chemotherapy.

The data collection process was done with RotatorSurvey, a software application for the Windows environment.

### Biopsy processing

For each procedure carried out, a biopsy-order-sheet-template was completed with relevant information for pathological analysis (breast or axilla, laterality, type of imagenological lesion, image-guided method, needle gauge, size, percentage of lesion removed by the needle biopsy device, BI-RADS classification, patient history and relevant observations).

Tissue samples were processed according to the American Society of Clinical Oncology and the College of American Pathologists Guidelines Recommendations. Samples were fixed in 10% neutral buffered formalin for 6–72 hours. Afterwards, alcohol-xilol and paraffin-embedded, 3-μm-thick histological sections were performed. A total of four to six cuts were obtained for each paraffin block. If the samples were associated with microcalcifications before the pathological processing of the tissue cylinders, a radiological control confirming the presence of microcalcifications was made. The biopsy interpretation was performed by breast pathologists with 5–25 years’ experience. PBLs were diagnosed and classified according to the histopathological criteria established by World Health Organization (WHO) [8, 17]. When the WHO criteria could not be established, the sample was classified as ‘Papillary lesion with suggestive changes of atypia’. In all of these cases, we performed surgical excision given the possibility of association with neoplastic proliferations [18–20]. This was done when partial or extensive sclerosis of a papillary lesion was observed either with or without atypia.

### Biopsy result management

A biopsy was considered **concordant** when the pathological evaluation provided an acceptable explanation for the imaging findings and **non-concordant** when the benign histopathological findings did not explain the imaging observed [21–23]. An **upgraded** was defined as a lesion that was benign (with/without atypia) on percutaneous needle biopsy but showed malignancy (*in situ* or invasive) in excision [14, 23] or during the imaging follow-up.

If the histopathological diagnosis of percutaneous biopsy resulted on benign lesions, concordant with the imaging and without any surgical indication, imaging follow-up in 6 or 12 months’ period was recommended. For core biopsies cases with atypical or suspicious alterations results in the pathological report or non-concordant pathologic result with the image, a second biopsy with a vacuum-assisted device was indicated for lesions less than 3 cm in size. For larger suspicious or non-concordant lesions, surgery was recommended. Surgery was recommended for all malignant lesions.

The identification of the residual tumour or the tumour bed was guaranteed for nonpalpable lesions, prior to surgery. The area of interest was localised using an imaging wire-guided localisation method [24]. Then, the intraoperative assessment of the breast surgical specimen was performed by imaging and pathological analysis, thus identifying the residual lesion, tumour bed and tumour-free margins Assessment of a palpable mass included radiologic and pathologic evaluation.

The purpose of the intraoperative radio-pathologic evaluation was to guarantee during the surgery the complete removal of lesion/tumour bed and tumour-free margins. The intraoperative assessment was never performed with diagnostic intentions**.**

### Statistical analysis

Data analysis was carried out with statistical software SPSS version 23, using descriptive statistic through squares, graphics, media, standard deviation, absolutes and relative values; inferential statistic that uses the Chi-Square statistical homogeneity test for the qualitative variables, the Kappa Test to determine the concordance of percutaneous biopsy and surgical biopsy, and sensibility, specificity, positive predictive value and negative predictive value of the diagnostic test based on percutaneous biopsy. Significance was stablished for the statistical test if *P* <0.005.

## Results

From March 1996 to December 2017, we processed 7,920 biopsies on 6,702 patients. A total of 305/7920 (3.85%) papillary lesions were diagnosed 2.38% (189/7920) benign and 1.47% (116/7920) malignant, all in women. Only 136 biopsies on 130 patients with papillary lesions met the inclusion criteria: 94 biopsies for benign papillary lesions (75 follow-up patients, 19 patients underwent surgery) and 42 biopsies for malignant papillary lesions. The distribution of cases according BI-RADS categories and its correlation with histological diagnosis and clinical behaviour are outlined in Table 1.

The mean age of patients with benign lesions was 59.37 years and with malignant lesions 63.0 years (range, 27–91 years). The imaging determined mean size was: benign lesions follow-up group 10.61 mm (range, 4–55 mm); benign lesions surgery group 17 mm (range, 4–50 mm) and malignant lesions surgery group 22.56 mm (range, 6–115 mm).

Most biopsies 123/136 (90.44%) were ultrasound guided and the remaining biopsies 13/136 (10.22%) were stereotactic guided. A total of 105 (77.20%) biopsies were performed with vacuum-assisted device and 31 (22.79%) with core biopsy. Although vacuum-assisted biopsies were performed with different probes (7G, 8G, 9G, 11G and 12G), most of them (44.12%) were done with 8G probes. All core biopsies were performed with 14-gauge needle and automatic gun.

The goal of the sampling with a vacuum-assisted device was the complete removal of the image that motivated the biopsy or at least most of them. Complete removal of the image occurred in 97.93% of cases. Sampling was partial only in 2.06% cases. Between 60% and 80% of the image was obtained in those with partial vacuum-assisted biopsy. The mean samples obtained with 14G needles were: 6.78 samples (range, 4–12) and with vacuum-assisted devices 14.67samples (range, 5–92).

The most common mammographic images were: mass not circumscribed 64(47.06%), mass not circumscribed with microcalcification 13(9.56%) and microcalcification alone 9(6.62%). There were 34(25%) lesions not visualised in the mammogram (Table 2). Sonographic appearance of lesions included: solid masses 75(55.15%), complex cystic and solid with and without microcalcification 48(35.3%). Eleven lesions (8.08%) were not visualised at ultrasound (Table 3).

Histological findings of benign lesions (follow-up group) are outlined in Table 4. Clinical and Imaging follow-up were done in 75/94(79.78%) patients with benign papillary lesions or papillary lesion with morphological changes suggestive of atypia (range, 6–217 months; mean follow-up, 62.7 months). All these biopsies were taken with the vacuum-assisted device (mean size: 10.63 mm) and complete removal of lesion occurred in 98.6% of the biopsies. Among these lesions, only one (1.33%) had an upgraded diagnosis after 3 years of follow-up, resulting in invasive papillary carcinoma diagnosed by ultrasound-guided CNB. For this case, pathologists performed a retrospective examination of the specimen initially diagnosed as a benign papillary lesion without atypia (6-mm mass, vacuum-assisted biopsy with 8G needle and sonographic guidance) and a misdiagnosed was confirmed corresponding this previous diagnosis to a PDCIS.

Surgical excision was performed on 20.21%(19/94) of the patients with benign papillary lesions. Nine patients (47.37%) underwent surgery because of surgeon´s recommendation, 9(47.37%) patients because of pathologist’s recommendation [papillary lesions with suggestive changes of atypia 4(21.05%), papillary lesion with atypical ductal hyperplasia 3(15.79%) and partially sclerosing papillary lesion 2(10.53%)] and 1(5.26%) patient because radiologist´s recommendation. Of these group of patients with surgical excision, 63.16%(12/19) were benign lesions without atypia and 36.84%(7/19) showed atypical changes according to the pathologists.

Table 5 shows the correlation between pathologic findings in patients as a result of a percutaneous biopsy and the final histologic result after surgery recommendation. All the patients with benign papillary lesion as a result of the percutaneous breast biopsy remained benign in the surgical biopsy. In the group with atypia, there was a correlation in all but two cases in which the surgery results were upgraded to a malignant disease.

If we consider the total of patients with benign lesions as a result of percutaneous biopsy (75 in the follow-up group plus 19 in the surgery group), the rate of underdiagnoses (two upgrades and one misdiagnosis) was 3.19%(3/94). If we take into account, the upgrades only the percentage would be (2.12%).

### Concordance between the results of percutaneous biopsy and surgical biopsy

The concordance between the results for percutaneous biopsy and surgical biopsy in patients with papillary lesions who underwent surgery was evaluated through the Kappa test. It measures the concordance between diagnostic methods. The valuation of the Kappa index, as an estimator of the strength of concordance between variables, was based on the following scale: <0.20: poor; 0.21–0.40: weak; 0.41–0.60: moderate; 0.61–0.80: good and 0.81–1.00: very good.

The results of this test indicate that there is a concordance between the results. Thus, the Kappa value of 0.753 indicates that the concordance between the results of the percutaneous biopsy and the surgical biopsies of patients with papillary lesion who underwent surgery is good (*P* = 0.000). If we observe the main diagonal in Table 6, we can highlight that there is a concordance in 88.70% of cases.

The mean time between percutaneous biopsy and surgery for malignant lesions was 43.23 days (range, 8–113 days). For all malignant lesions (42/137), there was concordance between CNB diagnosis and histological diagnosis from the final surgical sample: 34 invasive papillary carcinomas; seven invasive ductal carcinomas. In only one case, a vacuum-assisted biopsy from a 20-mm mass, BI-RADS 4B, showing an infiltrating ductal carcinoma, the surgical specimen resulted in a DCIS. This case was concluded as a papillary carcinoma with a DCIS component. For malignant lesions, there were no misdiagnoses. There were changes of histological varieties in seven cases in which the malignant lesion persisted.

We would like to highlight the cases described below. There were seven patients for whom two biopsy procedures were performed. One patient showed a synchronic malignant lesion. For three of them, the biopsies consisted of asynchronous bilateral breast lesions, taken several years apart and, for three patients, the biopsies were taken on the same day. These six lesions were benign, even the one that underwent surgery.

One patient with a benign lesion (size: 12 mm; BI-RADS 5) belonging to the follow-up group showed a non-concordant result between the histological findings and a 14 G CNB. A second biopsy was performed for this patient with a vacuum-assisted device confirming the benign diagnosis. Another patient in this group developed a mass at the same site where the first biopsy was taken. A new biopsy was performed and the histological diagnosis showed that it corresponded to a benign papillary lesion. A biopsy (8G needle vacuum-assisted breast biopsy) taken from a patient with nipple discharge demonstrated a benign intraductal papillary lesion (12-mm nodule). Nipple discharge ceased after the biopsy.

Four patients had limiting factors for interpretation: associated sclerosis or marked sample fragmentation. One case with a non-concordant biopsy according to the radiologist´s criteria corresponded to a 20-mm nodule BI-RADS 5 (ultrasound-guided CNB, 14G needle) and histological result showed a benign papillary lesion.

### Sensibility and specificity of the diagnostic test

The diagnostic test is the percutaneous biopsy, which is defined by the type of cannula selected (core biopsy and vacuum assisted) according to the BI-RADS category classification and also the size of the lesion. 95.56% of general sensibility was shown for both patients that underwent surgery and had a diagnostic test through a percutaneous biopsy. For the core biopsy alone, the sensibility was 95.83%, and for the vacuum-assisted cannula alone, it was 95.24%. The general specificity was 100% and the same percentage for CNB or vacuum-assisted cannula.

The probability that a patient presents malignancy with a positive percutaneous biopsy positive predictive value (PPV) is 100% with any type of cannula. The probability that patients present a benign result given that the percutaneous biopsy was negative (NPV) is 89.47% using both. 85.71% for the CNB and 91.67% for the vacuum-assisted cannula (See Table 7).

## Discussion

The number of papillary lesions in relation to the total percutaneous breast biopsies is few. In the literature, up to 8.1% [5, 25, 26] is reported. In our data, they represented only 3.85% of all biopsies performed. However, they represent a challenge for the radiologist and the pathologist, at the time of diagnosing either benign papillary lesions without or with atypia and malignant, and for the surgeon to determine the best approach.

The alterations that result in histologic papillary lesions of the breast are easy to be detected by imaging methods but not all lesions that turn out to be a papillary lesion in pathology can be identified with a pathognomonic image in mammography or ultrasound. Thus, it is less possible to discriminate between a benign lesion with or without atypia or malignant lesions with an imaging method alone [4, 27–30]. The presence of solid intraductual or intracystic nodular lesions could be associated with papillary lesions, and the observation of associated microcalcifications, on mammography, which could suggest according to some authors, an atypical papillary lesion [31, 32]. In our case, complex cystic and solid lesions represented only 35.03% (Table 3) of the papillary lesions seen on ultrasound. The rest of the cases were purely histopathological findings not suspected in the images.

Therefore, the diagnosis of papillary lesions is mainly histological. The diagnostic approach once an image lesion is detected (palpable or not) begins with awarding a BI-RADS category. In some cases, depending on the degree of suspiciousness, a percutaneous biopsy procedure should be performed with the guidance of an imaging method.

Since we started as an institution in 1996, we have worked with expert breast pathologists and, based on the percutaneous diagnostic tools, we came up with a working protocol that has given us greater confidence and security in histological results. It also helps us with decision making concerning surgical procedure or imaging follow ups with greater confidence. As a result of this, we consider that the choice of the type and thickness of the cannula must depend more on the size and the degree of suspicion of the image than the cost of it. In many cases, a second biopsy or a diagnostic surgery would be more expensive.

It is the breast radiologist´s decision, having observed a lesion in an imaging series, to choose the imaging guiding modality and the cannula to be used [33].

That is why, once the histological diagnosis of a papillary lesion is received, after a percutaneous biopsy, it is essential to evaluate how these patients will be followed in order to define who will be observed and who will undergo surgery.

It should be taken into consideration that the PPV of percutaneous biopsy is high for both benign and malignant lesions [26]. In our data, we obtained a PPV of 100% for biopsies taken with both cannulas (core and vacuum assisted). For patients that required surgery with benign papillomas or papillomas with atypia, percutaneous biopsy showed an average 95.56% of sensitivity for the vacuum-assisted device. The specificity was 100% with both cannulas. The probability of a patient presenting a benign result due to the fact that the percutaneous biopsy was negative (NPV) is 89.47% with both cannulas and 85.71% for the core biopsy. It was also 91.67% for the vacuum-assisted biopsy.

In this series, all the lesions diagnosed as malignant in the percutaneous biopsies, with any cannula, were confirmed malignant in the surgical biopsies with a coincidence of 100%. Therefore, the biopsy was used for definitive surgical planning. We had a case in which the percutaneous biopsy was diagnosed as infiltrating papillary carcinoma and the surgical specimen reported DCIS in relation to the previous biopsy site, interpreting the case as an infiltrative papillary carcinoma removed by the vacuum-assisted device (in which we obtained 100% of the lesion/image) with an associated perilesional DCIS that was identified in the surgical specimen.

Shin *et al* [34] published a small series in 2008 using a vacuum-assisted device and showing rigorous advantages over a core device. They found that CNB gave a sensitivity of 28% and specificity of 100%, whereas both sensitivity and specificity were 100% with vacuum-assisted biopsy. Although the number of vacuum-assisted biopsied lesions were small, the vacuum-assisted biopsy was more accurate than CNB in the diagnosis of papillary lesions and PPV of 100%, and NPV of 100% for the diagnosis of malignant papillary lesion [34]. A few years later, Chang *et al* [35] conducted a prospective study with the goal of determining the rate of malignancy after surgery in papillary lesions initially diagnosed at ultrasound (US)-guided 11-gauge vacuum-assisted breast biopsy. Their findings suggest that surgical excision may not be required for benign papillomas diagnosed after 11 gauge ultrasound-guided vacuum-assisted breast biopsies.

If we consider both the views of a breast trained radiologist and pathologist, the diagnosis of a malignant papillary lesion cannot represent a diagnostic problem when the sample is obtained through a percutaneous biopsy and the surgeon can plan the appropriate treatment in this group of patients. In our case, we had a good correlation between percutaneous biopsies and surgical biopsies, only presenting an upgrade of 3.19%. Even though it was not measured in our study, we believe expertise is of the greatest importance and of course the evaluation of warning signs by a dedicated breast pathologist. The importance of the expertise of breast pathologists was reported by Jakate *et al* [3] They concluded that with accurate pathologic assessment and radiologic-pathologic correlation, the upgrade rate of benign papilloma to malignancy can be minimised significantly. In a study published by Shiino *et al* [28], they observed that 41% of intra ductal papilloma on needle biopsy upgraded to carcinoma on surgical excision specimens, the core biopsies were performed with a 16-gauge needle guided by ultrasound or with stereotactic vacuum-assisted 10–11-gauge biopsies. Richter-Ehrenstein *et al* [29] studied 151 cases in which papilloma was diagnosed on core biopsy or surgical excision, the core biopsies were taken by stereotactic vacuum-assisted large gauge (11G) biopsy or by ultrasound-assisted automated (14G) CNB. The upstage rate of intraductal papillomas without atypia on CNB was 8.9%.

On the other hand, Pareja *et al* [36] recently published that in a group of 171 biopsies resulting from benign papillary lesions without atypia using CNBs observed a 2.3% rate of upgrading to carcinoma in excision, confirming that the upgrade rate with a radiologic-pathologic concordant is low. It has been observed that using vacuum-assisted devices, it is possible to obtain greater volume materials than core biopsies, and it could have lower upgrade rates [12, 32]. While we consider more the percentage of samples taken, in relation to the size of the imaging lesion, some publications considered the amount of cores [37]. Our goal with the vacuum-assisted device is to take the whole lesion/image without pretending that this is a surgical treatment [33]. However, a benefit of this type of device is to contribute to a more accurate diagnosis [38]. Seely *et al* [14] in a series of 107 patients observed a lower but not statistically significant upgrade rate of malignancy and atypia with the use of the 10–12 G vacuum-assisted breast biopsy as compared with 14G CNB and found the odds of an upgrade to malignancy to be 5.5 times higher with a 14 G needle than with vacuum-assisted breast biopsy.

If we evaluate the cases in our series of benign papillary lesions without atypia, we observe that the average size is 10.61 mm, and the biopsy was performed with a vacuum-assisted device with the complete removal of the lesion/image in most cases. The average size is small with a significant sample lesion removal. Symbol and Ricci [39], demonstrated a statistically significant relationship between lesion size >12 mm and increased proportion of atypia (*P* = 0.008).

In our data, when we have found a pathological report of benign papillary lesion and we achieved the removal of the lesion/image with the vacuum-assisted device our recommendation was imaging follow ups as recommended by several authors [12, 32, 38, 40]. On the other hand, Wang *et al* [23] presented a larger percentage (25%) of benign or atypical papillary lesions diagnosed on CNB upgraded in the final excisional examination in a primarily African–American patient’s urban population proposing that the patient’s ethnic group should be taken into account to decide the guidance. Wen and Cheng [41] conducted a meta-analysis to calculate a pooled estimate for the underestimation of malignant breast papillary lesions in non-malignant lesions at CNB and to identify variables associated with this underestimation. The median percentage of underestimation for the 34 studies was 15.7% (95% CI: 12.8%–18.5%). They concluded that the high rate of underestimation indicates that surgical excision should follow the diagnosis of atypical breast papillary lesions at CNB, and imaging follow-up is reasonable for patients with benign papillary lesions at CNB.

Among the cases studied, with the exception of a patient with misdiagnosis, which will be discussed later, there was no change in the diagnosis during the follow-up of patients with benign papillary lesions by 62.7-months, presenting lesions of 10.61-mm of average size. We took all the samples with vacuum-assisted device obtaining in almost all cases 100% of image. For the authors Wen and Cheng, there is no significant difference between a lesion that is smaller than 13.5 mm and one with a larger size but vacuum-assisted biopsy is considered more accurate than core-needle biopsy in diagnosing papillary lesions [41].

In the evaluation of our upgraded cases (3), we found that one of them was considered a misdiagnosis in the histopathological diagnostic criteria. This case was associated with DCIS from the beginning according to the retrospective evaluation. It was handled by a temporary pathologist who did not note the malignant lesion at that time. Another case was initially diagnosed as HAD, resulting in a papillary DCIS with a 1-mm IDC in the adjacent breast tissue in the mastectomy specimen. It was a case of underdiagnosis by sampling and not by histopathological diagnostic criteria. The third case was a partially sclerosed papillary lesion in core biopsy that resulted in a papillary infiltrating carcinoma in the definitive surgical specimen. In the past two cases, the recommendation of the expert pathologist was the removal of the lesion with free margins for diagnostic conclusion to be done in the surgical specimen.

It is interesting to note that in studies in which it has been attributed to premalignant lesions as atypical ductal hyperplasia among others, underdiagnosis percentages of up to 52%, have been diagnosed in percutaneous biopsy with core biopsy needle, without describing fundamental information such as the size and extent of the lesion [42, 43]. In our case, the pathologist always had valuable information such as the size and the percentage of material obtained of the lesion/image, which allowed them to estimate the representativity of the evaluated sample and to make recommendations of removal when it was truly pertinent in order to avoid underdiagnosis.

Another valuable factor for the pathologist was to recognise the role of the fragmentation of this type of material and the presence of sclerosis. These characteristics limit and hinder the diagnostic interpretation of papillary lesions, being aware of that helps the pathologist to minimize the possibility of error by a more accurate interpretation. In all the cases that these two characteristics were found in, the percutaneous biopsy was subjected to compatibility diagnosis. Thus categorised as ‘papillary lesions with or without suggestive changes of atypia’ or partially sclerose papillary lesion if it was the case, which according to the size, the imaging extension of the lesion and the imagenological-histopathological concordance, the pathologist recommended surgical excision for a complete histopathological study and final diagnostic conclusions.

It should be noted that the fragmentation in this type of lesion makes it difficult sometimes to apply the suggested diagnostic criteria to distinguish between a papillary lesion with atypical ductal hyperplasia versus papillary lesion with DCIS [8]. Likewise, in the evaluation of loose and disintegrated epithelial fronds of very few millimetres (1–2 mm or smaller), it is difficult to assess the real extension of monomorphic areas, especially when dealing with lesions with low nuclear grade.

In the data presented by Pareja *et al* [36], the fragmentation of papillary lesions was the only histological feature associated with upgrade. Four papillary lesions upgraded were fragmented, but 77 fragmented papillary lesions had not been upgraded. Nevertheless, the analysis or histopathological examination of papillary lesions is complex in many cases, both in core biopsy samples and in a surgical specimen [12]. Epithelial displacement is also described among the difficulties in the histological evaluation of papilloma in percutaneous biopsies samples [44].

The immunohistochemistry had a limited value in the diagnostic process because in all cases, it was essential to recognise the monomorphic zones of atypia and to categorise them as atypical ductal hyperplasia or intralesional DCIS, according to the diagnostic criteria of WHO [8]. Therefore, in all those cases associated with sclerosis, we prefer to evaluate the peripheral relation of a lesion with the adjacent breast parenchima to certainly establish its possible invasive character.

Due to the fact that in our data, there were very few underdiagnosis cases, it is not possible for us to define the value of the type of cannula. However, our results show very well our criteria and diagnosis in the management of papillary lesions. It allows us to point out the outstanding value of the percutaneous type of biopsy selection and pathological interpretation of the sample. It is done by a breast specialised radiologist and pathologist to establish recommendations in order to decide the definitive conduct.

As papillary lesions are uncommon in breast pathology, all the variables shown in the literature should be considered in a multi-centre controlled study: the approach of the imaging, the selection of the type of biopsy, the follow-up management and all others. This is similar to what has been done with low-risk DCIS [45] and also high-risk breast lesions [46].

## Recommendations

It is difficult to predict exclusively by imaging all papillary lesions and it is less possible to discriminate with imaging methods between a benign lesion with or without atypia or malignant lesions. The diagnosis of a papillary lesion is mainly histological.

If you have detected an image classify it using a BI-RADS category. If a biopsy is required, we recommend the following:

- A percutaneous breast biopsy procedure should be performed with the guidance of an imaging method.
- It is recommended that an expert breast radiologist and a pathologist are on the diagnostic team.
- It is important to have a working protocol (algorithm) that gives good confidence and security in histological results after a percutaneousbreast biopsy.
- If a histological diagnosis of a papillary lesion is received:
- To choose between surgical procedures or follow up, the team has to consider: 1) the representativeness of the sample; 2) the degree of suspiciousness of the image and 3) and the cost effectiveness between a possible second biopsy or a diagnostic surgical biopsy.

## Conclusion

In conclusion, the algorithm proposed in this paper for the management of PBLs includes, on one hand, a trained radiologist who decides the type of cannula and guiding image biopsy (taking into account the BI-RADS category and the size of the lesion) and the percentage of the sample taken. On the other hand, the adequate diagnostic criteria of a trained breast pathologist is also needed. An adequate concordance between the image and the pathology results are of utmost importance in cases in which a benign papillary lesion results with or without atypia. All these considerations significantly reduce the possibilities of upgrading favouring the decision making between follow-up or surgery.

## Conflict of interest

No financial or commercial interests to disclose.

## Figures and Tables

**Table 1.** Distribution according to bi-rads category and its correlation with histological diagnosis and clinical behavior.

BI-RADS	PBL			No. of biopsies
	Benign* (Follow-up)	Surgery**		
		Benign	Malignant	
4a	55	10	9	74
4b	15	3	4	22
4c	3	4	12	19
5	2	2	17	21
No. of biopsies	75	19	42	136

* patients who were followed up with benign histological results by percutaneous biopsy

** patients who were followed up with benign histological results by percutaneous biopsy

**Table 2.** Correlation between mammographic image and histological finding.

Stereotactic images	Benign		Malignant	Total	%
	Follow-up	Surgery	Surgery		
Mass not cicumscribed	26	11	27	64	47.06
Mass not cicumscribed with microcalcifications	3	1	9	13	9.56
Circumscribed mass	2	1	4	7	5.14
Circumscribed mass with microcalcifications	3	0	0	3	2.20
Architectural distortion	2	0	0	2	1.47
Architectural distortion with microcalcifications	0	1	0	1	0.74
Asymmetric breast tissue	1	0	0	1	0.74
Microcalcifications	8	1	0	9	6.62
Not visualised lesions	28	4	2	34	25
Mammography not performed	2	0	0	2	1.47
Total	75	19	43	136	
%	54.74	13.87	31.39		100

**Table 3.** Correlation between ultrasonographic images and histological findings.

Ultrasonographic Images	Benign		Malignant	Total	%
	Follow-up	Surgery	Surgery		
Solid lesion	29	10	36	75	55.15
Solid lesion with microcalcifications	2	0	0	2	1.47
Complex cystic and solid	34	7	6	47	34.56
Complex cystic and solid with microcalcifications	0	1	0	1	0.74
Not US visualised lesions	10	1	0	11	8.08
Total	75	19	43	136	**100**

US: ultrasound

**Table 4.** Percutaneous biopsy on benign lesions follow-up group. Histological findings.

Histological findings	*N*	%
Papilloma	70	93.33
Intraductal papiloma with focal ADH	1	1.33
Intraductal papiloma with suggestive changes of atypia	1	1.33
Multiple papillomatosis	3	4
Total	75	100

**Table 5.** Correlation between pathologic findings from percutaneous needle biopsy and the final surgical histologic results on patient with surgery recommendation.

Pathology finding from percutaneous needle biopsy	Pathology finding from surgical biopsy	*N*	%
Papillary benign lesion	Papillary benign lesion	11	57.89
Papillary lesion with suggestive changes of atypia	Intraductal papilloma with one focus of ADH*	2	10.53
Papilloma with ADH*	Focal ADH* in adjacent breast tissue	1	5.26
Papilloma fragmentedwith ADH multifocal	Invasive ductal carcinoma (Focus 1 mm)	1	5.26
Partially sclerosed intraductal papilloma with suggestive changes of atypia	Partially sclerosed intraductal papilloma	1	5.26
Partially sclerosed intraductal papilloma	Partially sclerosed intraductal papilloma	1	5.26
Partially sclerosed papillary lesion	Intracystic papillary carcinoma	1	5.26
Intraductal papilloma with suggestive changes of atypia	Juvenile papillomatosis with two focus of ADH	1	5.26
**Total**		19	100

(*) ADH: atypical ductal hyperplasia

**Table 6.** Concordance of results of percutaneous biopsy and surgical biopsy in patients with papillary lesion who underwent a surgery. Ceclines. Period from April 1996 to December 2016.

Result of percutaneous biopsy	Result of surgical biopsy				Kappa	P
	Benign	Benign with ADH	Benign with ESA	Malignant		
	**n (%)**			**n (%)**		
Benign	10 (16.13%)	1 (1.61%)	0 (0.00%)	1 (1.61%)	0.753	0.000*
Benign with ADH	0 (0.00%)	1 (1.61%)	1 (1.61%)	1 (1.61%)		
Benign with ESA	1 (1.61%)	2 (3.26%)	1 (1.61%)	0 (0.00%)		
Malignant	0 (0.00%)	0 (0.00%)	0 (0.00%)	43 (69.35%)		

Note:

* there is a significant concordance between the results of biopsies *P* <0.05; based on Kappa test

ADH: atypical ductal hyperplasiaESA: elements suggesting atypia

Source: Own done

**Table 7.** Parameters of diagnostic test in patients with a papillary lesion that underwent surgery. Ceclines. Period from april 1996 to december 2016.

Type of cannula	Parameters of diagnostic test			
	Sensibility	Specificity	VPP	VPN
Core	95.83%	100.00%	100.00%	85.71%
Vacuum-assisted	95.24%	100.00%	100.00%	91.67%
General	95.56%	100.00%	100.00%	89.47%

Note: VPP = value of positive prediction VPN = value of negative prediction

Source: own done
